# Serial Monodomy in the Gypsy Ant, *Aphaenogaster araneoides:* Does Nest Odor Reduction Influence Colony Relocation?

**DOI:** 10.1673/031.010.19501

**Published:** 2010-11-08

**Authors:** Terry McGlynn

**Affiliations:** Department of Biology, California State University Dominguez Hills, 1000 E. Victoria St., Carson, CA 90747

**Keywords:** army ant, colony size, emigration, nest architecture, olfaction, ventilation

## Abstract

Serial monodomy is the nesting behavior in which a colony of animals maintains multiple nests for its exclusive use, occupying one nest at a time. Among serially monodomous ants, the availability of unoccupied nests reduces the probability and costs of army ant attacks. It has been proposed that nest odors mediate serial monodomy in the gypsy ant, *Aphaenogaster araneoides* Emery (Hymenoptera: Formicidae), and that colonies avoid returning to previously occupied nests that harbor colony odors. To evaluate this hypothesis, the odors inside the nests of *A. araneoides* colonies were experimentally reduced through ventilation; the nest movement behaviors of treatment and control colonies were compared. Odor reduction was found to have increased the frequency of movements into and out of the treated nest, without a change in the total occupation duration in the treated nest. Nests with a more open architecture that permitted natural flow of air were reoccupied more quickly than nests with smaller nest entrances. In summary, the openness of the architecture of *A. araneoides* nests and the ventilation of air through nests alters the use of these nests. These findings further support the working hypothesis that nest-bound odors mediate the pattern of serial monodomy in *A. araneoides.*

## Introduction

The life history of many colonial organisms features regular nest relocation events. Colonies incur costs and risks when moving among nests (Smallwood 1982a), and the maintenance of unoccupied nests is expensive. The potential causes and advantages of nest relocations vary greatly with the attributes and environments of each species in question The existence of this expensive habit calls for an explanation.

Polydomous ants occupy multiple nests simultaneously, and may maintain multiple nests to reduce the costs of encounters with aggressors and predators. *Cataglyphis iberica* nests are routinely attacked by colonies of *Camponotus foreli*, and Dahbi et al. (2008) suggested that emigration to preexisting nests facilitates escape from *C. foreli.* The polydomous meat ant, *Iridomyrmex purpureus* enhances its foraging efficiency via polydomy (van Wilgenburg and Elgar 2007a), but also experiences a lower cost of attack by predatory echindas as a consequence of polydomy (van Wilgenburg and Elgar 2007b).

The use of multiple nests also occurs in serially monodomous colonies. Among serially monodomous ants, there has been no evidence produced to support an association with competition, territoriality, or resource limitation (McGlynn et al. 2004). On the other hand, the maintenance of unoccupied nests by serially monodomous colonies is potentially a mechanism to reduce the cost of predation.

Droual (1983a) proposed that the existence of multiple maintained nests may serve as an emergency evacuation system during army ant attacks. Investigations into the ants *Pheidole hyatti* and *P. desertorum* support this hypothesis, and it may be possible that alternating use of nests reduces the probability of attack (Droual 1983b). Droual (1984) found that colonies of *P. desortorum* under attack from army ants experienced greater brood survival rates when multiple nests were available for rapid emigration.

The adaptive significance of serial monodomy has been further studied in the Central American gypsy ant, *Aphaenogaster araneoides* Emery (Hymenoptera: Formicidae). Colonies of *A. araneoides* relocate their nests an average of once per week, maintaining from 2–6 nests over the course of one month (McGlynn et al. 2004). Larger colonies move more often than smaller colonies, and colonies with more brood maintain more nests. Relocation behaviors do not respond to ambient or manipulated changes in food abundance (McGlynn 2006). Among a host of biotic and abiotic factors, nest occupation and relocation behaviors are associated with the size of the nest opening and ambient vapor pressure deficit, and colonies apparently use odor-based cues to trigger nest evacuations seconds prior to raids of predaceous army ants (McGlynn et al. 2002; McGlynn 2007). McGlynn (2007) proposed that nest quality is increased when odors are reduced, as nest odors may attract army ants or inhibit their detection.

Nests may retain volatile odors for extended periods, even when unoccupied. Nest architecture is a major influence on gas exchange, and gradients in gases occur even at relatively shallow depths (Tschinkel 2004). The structure of *A. araneoides* nests features a consistent cross-sectional area along the depth profile up to the nest entrance and this cross-sectional area of nest tunnels is a major determinant gas exchange rates. Nests with narrower entrances and tunnels are more likely to retain odors (Withers 1978).

In the present study, I test whether colonies of *Aphaenogaster araneoides* will more heavily utilize nests that harbor fewer volatile odors is tested. This idea is approached by comparing three nest relocation metrics among colonies with different size nest openings and with those that have been experimentally ventilated to reduce odors harbored in the nest.

## Methods and Materials

All field research was conducted in an old growth tropical rain forest within La Selva Biological Station, located in the Atlantic lowlands of northeastern Costa Rica. Work was carried out between May and July 2008. More information about La Selva is available at www.ots.ac.cr.

Nests of *A. araneoides* were located within 20 m of the Camino Experimental Sur trail between the trail markers 300 and 800 meter mark. Nests were located by direct searching and by attracting foragers to bait and following them back to their nests. Every 24 h, between 07:00 and 12:00, every nest was observed to assess for the presence of a colony within the nest. At the first detection of colony emigration, the marked nest was haphazardly assigned to either a treatment or control category in equal proportion.

Nests in the treatment category received an odor treatment regimen. In this regimen, 6 mm wide clear vinyl tubing was inserted into the nest for around 4 h of ambient ventilation with a portable battery-powered aerator pump at a rate of 850 cm3/min. The tube was inserted about 15 mm into the nest. This tube was appreciably smaller than the size of the nest entrance, and though the nest entrances were not altered, equivalent nest disturbances have been found to not affect nest relocation behavior (McGlynn et al. 2004). The tube remained within the nest for 24 h before removal. The control regimen was identical to the treatment regimen, except there was no ventilation of air through the tubing. After the tube was removed, the nest was monitored on a 24 h frequency for 10 d after the first observed reoccupation.

The size of the entrance to each nest was measured using the equation for the area of an oval with the maximum diameter measured to mm accuracy, and the diameter of the line perpendicular to the maximum diameter at its midpoint was measured to mm accuracy. The distribution of nest sizes was overtly bimodal and could not be corrected by transformation, and nest area sizes were distributed into two discrete size classes for analyses, small (1.5 to 2.5 cm2) and large (4.0 to 15.4 cm2). All statistical analyses were conducted in JMP 8.0.1 (SAS Institute, www.sas.com).

Three behavioral variables pertaining to nest movement were independently calculated. *Latency to reoccupation* was calculated in the number of days that had passed before the nest was reoccupied after the initial departure. This variable was calculated for all nests that were reoccupied after the termination of the treatment or control regimen for more than a single observation. Total *occupation frequency* was calculated as a ratio between 0 and 1, representing the fraction of days the nest was occupied out of the first 10d after the termination of the treatment or control regimen. Colonies demonstrated heterogeneity in the frequency in which they occupied, vacated, and reoccupied nests measured by *movement frequency.* This was calculated using the ratio of movement observations against the total duration of the days elapsed between initial reoccupation and final observation within the nest with a minimum of 6 days of observation. The effect of nest opening entrance area and treatment on latency to occupation, occupation frequency and movement frequency was evaluated using Generalized Linear Models.

## Results

**Figure 1.**
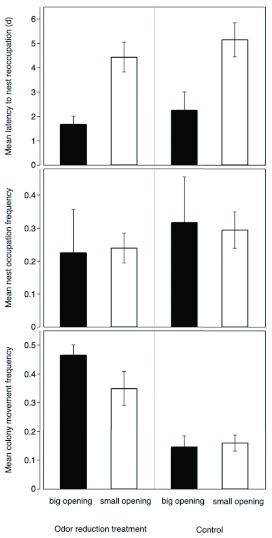
Nest movement behavior reflects differences in nest opening size and odor reduction treatment in *Aphaenogaster araneoides.* Nest occupancy was evaluated on a daily basis. Error bars represent standard errors; GLM analyses are presented in Table 1. High quality figures are available online.

The latency to nest reoccupation was significantly influenced by the area of the nest entrance opening, but not by the odor reduction treatment (Table 1, Figure 1). The nest occupation frequency was not affected by nest entrance size nor the odor reduction treatment, though odor reduction caused colonies to move to and from the focal nest more often (Table 1, Figure 1).

## Discussion

Both nest ventilation and nest opening size unambiguously altered the nest movements of *A. araneoides*, in directions suggesting that colonies more heavily use nests that are less likely to retain odors. These results are consistent with an earlier finding that colonies with experimental addition of colony odors decreased nest use by *A. araneoides* (McGlynn 2007). Interpreted in concert, the results reinforce the working hypothesis that serial monodomy in *A. araneoides* is structured, in part, by the retention of odors within nests.

The most striking find was that colonies were more apt to reoccupy a previously emigrated nest more quickly if the nest had a more open architecture. This typically would be seen as an enigmatic find, as wide open nests would typically be viewed as poor quality spaces for ant colonies as social insect colonies typically occur as ““fortresses”” (Oster and Wilson 1978). Because nests with large entrances were far more quickly reoccupied, this leads us to consider an adaptive function for the broad nest openings.

**Table 1.**
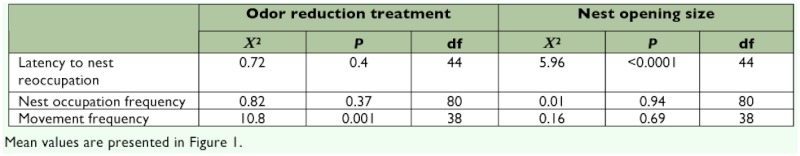
Results of Generalized Linear Model on the effect of odor reduction treatment and nest entrance size on three measures of nest movement behavior.

The nest architecture of all *A. araneoides* colonies is remarkably open, relative to nearly all other soil-nesting ants. An observer with a focused light can often peer into the uppermost chamber of the nest. It is reasonable to surmise that the open nest architecture is adaptive. The broad nest opening may serve to facilitate rapid evacuation —— which can be completed in less than five seconds in the face of army ant raid (personal observation). The large nest opening may also permit odors to evaporate from the nest more readily, though this would be more supported if nests subjected to air treatment experienced a reduction in latency to occupation and this did not occur.

Nest ventilation did have an effect on colony movements, in which treatment colonies moved into and out of treatment nests a greater number of times than control colonies —— without any increase in the cumulative amount of time spent in the focal nest. This enigmatic result does not indicate that colonies do not favor the treated nests; nests that are unfavored continue to be unoccupied (McGlynn 2007). The continued return to a nest is an indicator that ventilation enhances perceived nest quality, but it is unclear why the colonies simply remain in the treatment nests rather than come and go more frequently.

One possible explanation that is consistent with the findings is that colony life history influences the response to treatment. Specifically, it is possible that larger colonies are more sensitive to the potential accumulation of odors within nests. Larger colonies of *A. araneoides* move more frequently than smaller colonies (McGlynn et al. 2004). The observed result is consistent with the explanation that larger colonies are more sensitive to the odor treatment than smaller colonies. This is intuitively sensible as larger colonies are expected to accumulate more odors in their nests as a result of the greater number of individuals. This hypothesis may be tested by a new experiment on net ventilation that incorporates colony size as a new variable, ideally incorporating headspace analysis of nest-bound odors.

While army ant predation apparently plays a role in the maintenance of serial monodomy in *A. araneoides*, the findings of this study do not account for the broader phenomenon of serial monodomy and other nest relocation behaviors that are widespread in many lineages, including temperate *Aphaenogaster* species such as *A. rudis* complex spp. (Smallwood 1982b) *and A. senilis* (AvarguesWeber and Monnin 2009), which relocate frequently but are not heavily depredated by army ants. The capacity for nest relocation is found in many Myrmicine ants including *Aphaenogaster* (Smallwood 1982a; reviewed in McGlynn et al. 2004), and the adaptive value of nest relocations varies with the changing ecological theater of a given taxon.

In summary, the present findings cumulatively reinforce the working hypothesis that odors mediate, in part, the nest movements of *A. araneoides.* These findings lead the author to consider a new set of testable hypotheses about nest-bound odors that may be directly tested by conducting headspace analysis of odors bound within nests. It may be predicted that the concentration of volatile odors is positively associated with colony size and occupation duration, and negatively associated with time since occupation and nest opening size.
